# Effect of transcutaneous auricular vagus nerve stimulation on perioperative anxiety in patients undergoing radical mastectomy: study protocol for a randomized controlled clinical trial

**DOI:** 10.3389/fneur.2025.1682692

**Published:** 2026-01-12

**Authors:** Jiayi Tang, Ming Yao, Tong Wu, Huadong Ni

**Affiliations:** Department of Anesthesiology and Pain Research Center, The First Hospital of Jiaxing, The Affiliated Hospital of Jiaxing University, Jiaxing, China

**Keywords:** perioperative anxiety, protocol, radical mastectomy, randomized controlled trial, transcutaneous auricular vagus nerve stimulation

## Abstract

**Background:**

Breast cancer patients often experience significant concerns regarding surgery, which predisposes them to negative emotions such as anxiety, adversely affecting both surgical outcomes and postoperative recovery. However, research on interventions for perioperative anxiety remains limited. This study focuses on patients undergoing radical mastectomy to investigate the effects of transcutaneous auricular vagus nerve stimulation (taVNS) on perioperative anxiety, aiming to provide novel clinical strategies for its prevention and management.

**Methods:**

This prospective, double-blind, randomized controlled trial will enroll 202 eligible patients with radical mastectomy. Patients are randomly assigned to either the treatment group (taVNS group) or the control group (sham stimulation group). In treatment group, taVNS is administered 30 min before anesthesia induction and at the same time point daily for postoperative days 1–3, using specific stimulation parameters. The control group was given a sham stimulus. Primary outcome is the perioperative anxiety remission rate, which assessed by comparing post-intervention (after the final session) and pre-intervention (before the first session) scores in patients subjected to either full-course taVNS or sham stimulation. Secondary outcomes are Hamilton Anxiety Rating Scale (HAMA), Pittsburgh Sleep Quality Index (PSQI), 3-Minute Diagnostic Interview for Confusion Assessment Method-Defined Delirium (CAM-3D), Numerical Rating Scale (NRS), Fatigue, Resistance, Ambulation, Illnesses, and Loss of Weight (FRAIL) and heart rate variability (HRV).

**Discussion:**

The strength of this study lies in the adoption of taVNS as an intervention for perioperative anxiety and the establishment of a comprehensive efficacy evaluation system. taVNS may alleviate anxiety by modulating autonomic nervous function and restoring the balance between sympathetic and parasympathetic activity. This study aims to provide an innovative approach for mitigating perioperative anxiety, with the potential to improve patient outcomes and enhance quality of life.

**Clinical trial registration:**

https://www.chictr.org.cn/indexEN.html, Identifier ChiCTR2500106867.

## 1 Introduction

Breast cancer remains one of the most significant global health challenges. According to the latest report from the World Health Organization (WHO) International Agency for Research on Cancer (IARC), breast cancer ranks as the second most prevalent malignant tumor worldwide (1). China accounts for the highest number of breast cancer cases, representing approximately 18.4% of the global burden (2), which imposes substantial adverse impacts on public health and socioeconomic development in the country. Currently, the primary treatment strategy for breast cancer remains surgical intervention (3, 4). As a major negative life event, radical mastectomy can induce psychological distress such as anxiety and depression in patients. Previous studies have demonstrated that approximately 41.9% of breast cancer patients experience perioperative anxiety (5). Research indicates that compared to patients with malignancy alone, those with perioperative emotional disorders exhibit a 19% increase in mortality (6), along with elevated treatment-related adverse effects and diminished therapeutic efficacy. Consequently, early intervention for perioperative anxiety is of critical importance.

Currently, both pharmacological and non-pharmacological approaches are commonly employed in clinical practice to alleviate perioperative anxiety. However, anxiolytic medications may induce respiratory depression and prolong recovery time, thus their routine preoperative administration is not recommended (7). Non-pharmacological interventions, such as psychotherapy and self-regulation techniques, are being increasingly implemented in clinical settings. Additionally, non-invasive neuromodulation technologies have demonstrated considerable potential in mitigating perioperative anxiety in patients (8).

In 2000, Canadian scholar Ventureyra developed transcutaneous auricular vagus nerve stimulation (taVNS) based on auricular acupuncture theory, anatomical principles, and traditional vagus nerve stimulation techniques. Compared with conventional invasive approaches, taVNS demonstrates superior safety, lower cost, and greater operational simplicity, establishing itself as a prominent adjunctive therapy. The core mechanism of taVNS involves modulation of autonomic nervous system function and homeostasis, pioneering a novel therapeutic paradigm targeting the peripheral nerve-brain network for systemic regulation. Clinical breakthroughs have been achieved in treating insomnia, depression, and epilepsy (9). Heart rate variability (HRV), defined as the variation in time intervals between consecutive normal heartbeats, serves as a standardized method for assessing autonomic nervous function. This metric offers continuous, objective, sensitive, and noninvasive monitoring advantages (10, 11).

Currently, there is limited research on the application of taVNS in perioperative anxiety management. This study selected patients undergoing radical mastectomy for breast cancer to investigate the efficacy of taVNS in alleviating preoperative anxiety. The mechanistic underpinnings were further explored through HRV monitoring, aiming to provide novel insights for postoperative rehabilitation strategies.

## 2 Methods and analysis

### 2.1 Study design

**Figure 1:**
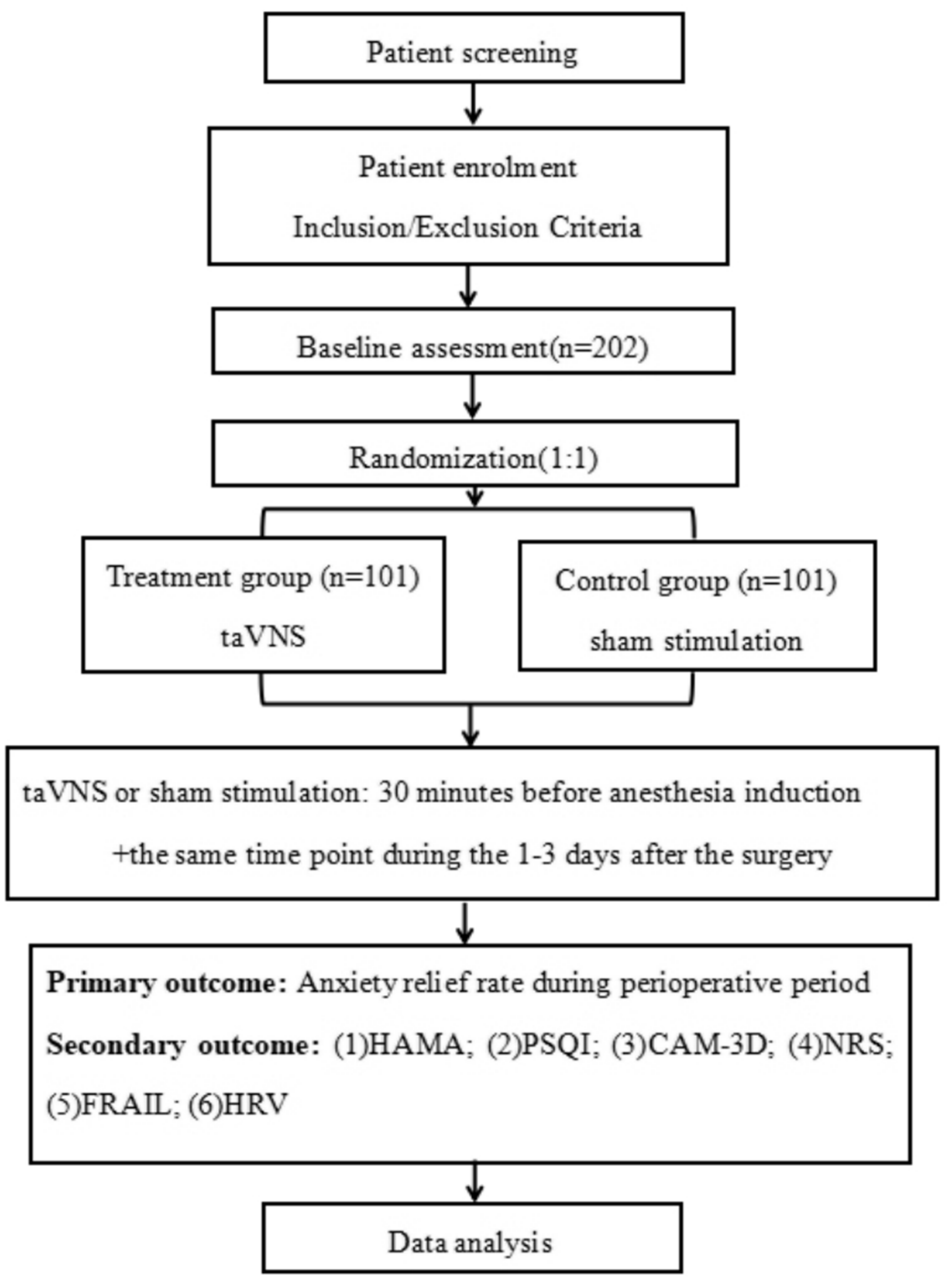
Flow chart of the study. taVNS, transcutaneous auricular vagus nerve stimulation; HAMA, Hamilton Anxiety Rating Scale; PSQI, Pittsburgh Sleep Quality Index; CAM-3D, 3-Minute Diagnostic Interview for Confusion Assessment Method-Defined Delirium; NRS, Numerical Rating Scale; FRAIL, Fatigue, Resistance, Ambulation, Illnesses, and Loss of Weight; HRV, heart rate variability.

This prospective randomized controlled trial will enroll 202 patients scheduled for radical mastectomy at our institution. Using a computer-generated randomization table, eligible participants will be allocated in a 1:1 ratio to either the treatment group (taVNS group) or the control group (sham stimulation group). The trial flow chart is shown in Figure 1. All participant characteristics and clinical outcomes will be documented using case report forms (CRFs). Data collection procedures are outlined in Table 1.

**Table 1:** Schedule of enrolment, interventions, and assessments.

Procedure	Study period						
	T0	T1	T2	T3	T4	T5	T6
Enrolment							
Eligibility screen	✓						
Informed consent	✓						
Allocation	✓						
Randomization	✓						
Interventions							
taVNS	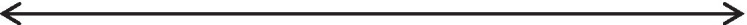						
Sham stimulation	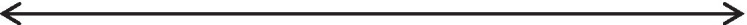						
Assessments							
HAMA	✓	✓	✓	✓	✓	✓	✓
CAM-3D			✓	✓	✓	✓	
NRS			✓	✓	✓	✓	
FRAIL				✓	✓	✓	
PSQI				✓	✓	✓	
HRV	✓	✓	✓	✓	✓	✓	
Adverse events	Throughout the study period until discharge						

taVNS, transcutaneous auricular vagus nerve stimulation; HAMA, Hamilton Anxiety Rating Scale; PSQI, Pittsburgh Sleep Quality Index; CAM-3D, 3-Minute Diagnostic Interview for Confusion Assessment Method-Defined Delirium; NRS, Numerical Rating Scale; FRAIL, Fatigue, Resistance, Ambulation, Illnesses, and Loss of Weight; HRV, heart rate variability. T0, before the initial taVNS or sham stimulation; T1, after the initial taVNS or sham stimulation; T2, 2 h after surgery; T3, 1 day after surgery; T4, 2 day after surgery; T5, 3 day after surgery; T6, 30 day after surgery.

### 2.2 Sample size

According to the Chinese Guidelines for the Prevention and Treatment of Anxiety Disorders, which define “a response rate ≥50% as effective and <30% as ineffective.” This study hypothesized response rates of 50% in the treatment group and 30% in the control group. With a statistical power of 0.80 and a significance level of 0.05, sample size calculation using PASS 15.0 software indicated that 91 patients will be required per group. Accounting for a 10% dropout rate, we plan to enroll 101 participants per group, totaling 202 subjects.

### 2.3 Inclusion criteria

Participants who meet all the following requirements will be enrolled:

(1)  Female patients aged 20–85 years.
(2)  Patients scheduled for elective radical mastectomy.
(3)  Preoperative patients with Hamilton Anxiety Rating Scale (HAMA) scores ≥7 measured before intervention.
(4)  Patients with elementary school education or above, and normal communication, hearing, and comprehension abilities.
(5)  Participants who voluntarily signed informed consent forms.
(6)  Patients classified as American Society of Anesthesiologists (ASA) Physical Status I–III.

### 2.4 Exclusion criteria

Participants who meet one of the following requirements will be excluded:

(1)  History of neurological disorders or psychiatric disorders (with drug treatment).
(2)  Severe cardiovascular or cerebrovascular diseases.
(3)  Contraindications for taVNS: recent auricular trauma, skin lesions, erythema, or infection in the auricular region; presence of implantable electrophysiological devices (e.g., cardiac pacemakers, stents, or prosthetic joints).
(4)  Chronic use of psychotropic medications (e.g., corticosteroids, antidepressants, or anxiolytics).

### 2.5 Criteria for discontinuation

During this trial, subjects experiencing the following conditions will be considered as dropout cases, and the reason and date of dropout will be recorded in a case report form.

(1)  Intraoperative blood pressure or heart rate could not be maintained, or severe intraoperative complications occurred (e.g., hemorrhagic/anaphylactic shock, cardiac arrest, etc.).
(2)  Patients who refused to undergo relevant scale assessments.
(3)  Incomplete HRV monitoring data.
(4)  Loss to follow-up.

### 2.6 Randomization and blinding

Participants will be randomly assigned in a 1:1 ratio to either the treatment group (taVNS group) or the control group (sham stimulation group) based on a computer-generated randomization table. Allocation information will be sealed in opaque envelopes and will remain concealed until the intervention be administered by investigators prior to surgery. Outcome assessors, data processing personnel, other healthcare providers, and patients will be blinded to treatment allocation.

## 3 Procedure

Participants will be screened from the elective surgery roster by the research team. At 30 min prior to anesthesia induction and at the same time point daily for postoperative days 1–3, patients will be subjected to either taVNS (treatment group) or sham stimulation (control group).

*Treatment group*: Electrical stimulation will be applied through electrodes placed on the cymba conchae. Following clip placement, taVNS will be administered for 30 min per session. The device will be activated with the following parameters:

- Waveform: Dense-disperse wave.
- Mode: Constant voltage.
- Frequency: 20 Hz.
- Pulse width: 0.2 ms.
- Intensity: Subthreshold current (maximally tolerated without eliciting sharp pain).

*Control group*: The electrodes will be positioned identically, and the current will be increased over 30 s until the participant reported a tingling sensation. Then reduced to a level just below this perceptual threshold. The device remain off for the subsequent 30 min to simulate the sham condition (12).

## 4 Outcome

### 4.1 Primary outcome

The primary outcome is perioperative anxiety alleviation rate in patients subjected to either full-course taVNS or sham stimulation before the first intervention and after the last intervention. The anxiety alleviation rate will be calculated as follows: (baseline score − post-intervention score)/baseline score × 100%.

Perioperative anxiety will be assessed using the Hamilton Anxiety Rating Scale (HAMA), a 14-item scale with a total score ranging from 0 to 56. The severity of anxiety will be categorized as follows: total score <7: no anxiety; total score 7–13: mild anxiety; total score 14–20: moderate anxiety; total score >20: severe anxiety.

Patient anxiety will be assessed before (T0) and after (T1) the initial taVNS or sham stimulation. Further assessments occurr at 2 h post-surgery (T2), following the intervention on postoperative days 1 to 3 (T3–T5), and at 30 day after surgery (T6).

### 4.2 Secondary outcome

#### 4.2.1 Clinical symptom assessment indicators

(1)  Hamilton Anxiety Rating Scale (HAMA) (T0 to T6).
(2)  Sleep quality score (T3 to T5): assessed using the Pittsburgh Sleep Quality Index (PSQI), with scores ranging from 0 to 21 (higher scores indicate poorer sleep quality).
(3)  Incidence of postoperative delirium (T2 to T5): evaluated using the 3-Minute Diagnostic Interview for Confusion Assessment Method-Defined Delirium (CAM-3D).
(4)  Pain score (T2 to T5): measured via the Numeric Rating Scale (NRS), ranging from 0 (no pain) to 10 (most severe pain).
(5)  Frailty score (T3 to T5): assessed using the Fatigue, Resistance, Ambulation, Illnesses, and Loss of Weight (FRAIL), with scores ranging from 0 (most robust) to 5 (most frail).

#### 4.2.2 Clinical examination assessment indicators

*Heart rate variability (T0 to T5)*: Heart rate variability (HRV) parameters, including low-frequency power (LF), high-frequency power (HF), the ratio of LF to HF (LF/HF), the standard deviation of normal-to-normal intervals (SDNN), the standard deviation of the average NN intervals extracted for each 5-min segment (SDANN), and the percentage of adjacent NN intervals differing by more than 50 ms (pNN50), will be measured using an autonomic nervous function analyzer (ZSY-1, Shenyang Weijin).

Under the assistance of nursing staff, HRV measurements will be obtained from conscious, seated patients during a standardized morning window (8:00 to 11:00). During the test, the patient maintain calm breathing while holding a sensor fixed in the palm for 5 min to obtain HRV data, which transmit to a computer for analysis.

## 5 Data collection and study safety

### 5.1 Data collection

Demographics and preoperative conditions: gender, age, body mass index (BMI), American Society of Anesthesiologists (ASA) physical status classification, HAMA score, and comorbidities (hypertension, heart disease) will be recorded.

Intraoperative parameters: intraoperative fluid volume, blood loss, urine output, surgical duration, postoperative extubation time, and time of admission to the post-anesthesia care unit (PACU) will be documented.

### 5.2 Study safety

Adverse events related and unrelated to taVNS will be recorded: skin erythema or rash, discomfort at the electrode site, dizziness, nausea, bradycardia, tinnitus, or hearing changes.

## 6 Statistical analysis

Statistical analysis will be performed using SPSS 23.0 (IBM Corp, Armonk, NY, United States). The normality of continuous variables was assessed using the Kolmogorov–Smirnov (K–S) test. Normally distributed data were expressed as mean ± standard deviation (SD). One-way analysis of variance (ANOVA) was employed for multiple group comparisons, while the independent t-test was used for pairwise comparisons between groups. Paired *t*-test was applied for within-group comparisons. Non-normally distributed data were presented as median (interquartile range) and analyzed using nonparametric rank-sum tests. Categorical variables were compared using the chi-square test. A two-tailed *p*-value <0.05 was considered statistically significant.

The primary outcome measure will be analyzed using the intention-to-treat (ITT) principle. Missing data for the primary outcome were handled using multiple imputation by chained equations under the missing-at-random assumption. The interpolation model includes all the analytical variables as well as any auxiliary variables that may be related to the detachment. We generated imputed datasets using the mice package in R. The results from the imputed datasets were combined using Rubin’s rules.

## 7 Discussion

To the best of our knowledge, this study represents the first systematic evaluation of the clinical efficacy and safety of taVNS for perioperative anxiety within the framework of a randomized controlled clinical trial (RCT) in China. The findings are expected to facilitate more effective management of perioperative anxiety, thereby improving patient outcomes and guiding clinical decision-making.

The autonomic nervous system (ANS), comprising the sympathetic nervous system and parasympathetic/vagus nerve, serves as a profound homeostatic regulatory system and constitutes a core component of emotional states. It reflects both vulnerability and resilience to psychological and physical health challenges (13, 14). Several disease-specific studies have demonstrated a close association between autonomic activity and emotional states such as depression and anxiety (15). Thayer and Lane (16) proposed that sympathetic activity serves as a determinant of emotional states, with the classic autonomic efferent pattern during emotional responses characterized by increased sympathetic activity coupled with decreased parasympathetic activity. In contrast, Jarrett et al. (17) suggested that negative emotional states (e.g., anxiety and depression) are exclusively associated with reduced parasympathetic tone. Consequently, the precise mechanisms underlying perioperative anxiety remain unclear, though they may involve disruption of ANS homeostasis.

The auricular branch of the vagus nerve represents the sole cutaneous branch of the vagus nerve. According to modern anatomical theory, the auricle is richly innervated, with nerve fibers predominantly concentrated in the cymba conchae, cavum conchae, and triangular fossa. Notably, the auricular concha region contains the only cutaneous vagal afferent fibers in humans. Experimental studies employing horseradish peroxidase (HRP) retrograde tracing of sensory neurons have untangled a potential neural pathway between the vagus nerve in the auricular concha region and the sympathetic ganglia innervating visceral organs. Furthermore, HRP labeling experiments have demonstrated that stimulation of the auricular concha region transmits signals to the nucleus tractus solitarius and medulla oblongata in the brainstem (18).

To address the limitations of implantable vagus nerve stimulation (VNS), including high procedural complexity, frequent complications, and substantial costs, transcutaneous auricular VNS (taVNS) was developed as a noninvasive alternative. This approach maintains therapeutic efficacy while reducing both risks and expenses (19). In 2010, Europe approved a taVNS device for the treatment of epilepsy and depression. Current research on taVNS efficacy has been conducted across multiple domains, including psychiatric disorders (e.g., depression, anxiety disorders, post-traumatic stress disorder), neurological diseases (e.g., epilepsy, migraine, stroke rehabilitation), and cardiovascular conditions. Recent mechanistic studies on taVNS suggest that its effects are not merely mediated by short-term electrochemical actions resulting from localized nerve fiber stimulation. Instead, taVNS exerts long-term regulatory effects through the neuro-endocrine-immune network (20).

This study employed a rigorous methodological strategy to enhance the reliability and validity of the research outcomes. First, randomization and blinding procedures were implemented to minimize potential biases and confounding factors. Second, a comprehensive set of endpoints was selected, including the primary efficacy indicator (perioperative anxiety relief rate) and multidimensional secondary endpoints—such as the HAMA, PSQI, CAM-3D, NRS, FRAIL, and autonomic nervous system monitoring HRV—to holistically evaluate the therapeutic effects in breast cancer patients undergoing radical mastectomy with perioperative anxiety. Notably, the study protocol innovatively incorporated HRV as an objective assessment tool to comprehensively evaluate physiological changes before and after anxiety relief. HRV, an internationally recognized and widely utilized noninvasive indicator for autonomic nervous system monitoring, reflects the balance between sympathetic and parasympathetic nervous activity as well as the complex interactions between the brain and cardiovascular system by detecting subtle variations in successive heartbeats (21, 22). HRV accurately captures changes in autonomic nervous system (ANS) function and balance. Previous studies have demonstrated that breast cancer patients with preoperative anxiety exhibit lower HRV after anesthesia induction compared to non-anxious patients (23). Additionally, breast cancer patients with a history of mood disorders display reduced autonomic flexibility following stress exposure (24), suggesting that HRV may serve as a potential biomarker for assessing negative emotional states such as anxiety and depression in cancer patients.

This study addresses the clinical challenge of perioperative anxiety by selecting patients undergoing radical mastectomy for breast cancer to investigate the effects of taVNS on alleviating perioperative anxiety. The autonomic nervous function was monitored through HRV analysis to explore the underlying mechanisms. The findings may provide novel insights for developing postoperative rehabilitation strategies.

## 8 Limitations

This study also has limitations. First, due to resource constraints, our assessment of anxiety relief was limited to the first three postoperative days. This timeframe is adequate for evaluating short-term therapeutic effects, but it may not fully capture long-term treatment outcomes. Second, the stringent inclusion criteria may restrict the generalizability of our findings to broader populations. Thirdly, the definition of a “subthreshold current” as the maximum intensity tolerated without eliciting sharp pain introduces substantial inter-patient variability that lacks standardization. This subjectivity represents a well-recognized challenge in non-invasive neuromodulation research. Therefore, real-world studies with extended follow-up and expanded patient populations will be conducted in the future.

## Trial status

Recruitment began in August 2025 and the approximate date when recruitment will be completed in August 2026. The study protocol was approved by the ethical committees in July 2025.
